# Characteristics of non-coplanar IMRT in the presence of target-embedded organs at risk

**DOI:** 10.1186/s13014-015-0494-5

**Published:** 2015-10-12

**Authors:** Klaus Bratengeier, Kostyantyn Holubyev

**Affiliations:** Department of Radiation Oncology, University of Würzburg, Josef-Schneider-Str. 11, 97080 Würzburg, Germany

## Abstract

**Background:**

The aim is to analyze characteristics and to study the potentials of non-coplanar intensity modulated radiation therapy (IMRT) techniques. The planning study applies to generalized organ at risk (OAR) – planning target volume (PTV) geometries.

**Methods:**

The authors focus on OARs embedded in the PTV. The OAR shapes are spherically symmetric (A), cylindrical (B), and bended (C). Several IMRT techniques are used for the planning study: a) non-coplanar quasi-isotropic; b) two sets of equidistant coplanar beams, half of beams incident in a plane perpendicular to the principal plane; c) coplanar equidistant (reference); d) coplanar plus one orthogonal beam. The number of beam directions varies from 9 to 16. The orientation of the beam sets is systematically changed; dose distributions resulting from optimal fluence are explored. A selection of plans is optimized with direct machine parameter optimization (DMPO) allowing 120 and 64 segments. The overall plan quality, PTV coverage, and OAR sparing are evaluated.

**Results:**

For all fluence based techniques in cases A and C, plan quality increased considerably if more irradiation directions were used. For the cylindrically symmetric case B, however, only a weak beam number dependence was observed for the best beam set orientation, for which non-coplanar directions could be found where OAR- and PTV-projections did not overlap. IMRT plans using quasi-isotropical distributed non-coplanar beams showed stable results for all topologies A, B, C, as long as 16 beams were chosen; also the most unfavorable beam arrangement created results of similar quality as the optimally oriented coplanar configuration. For smaller number of beams or application in the trunk, a coplanar technique with additional orthogonal beam could be recommended. Techniques using 120 segments created by DMPO could qualitatively reproduce the fluence based results. However, for a reduced number of segments the beam number dependence declined or even reversed for the used planning system and the plan quality degraded substantially.

**Conclusions:**

Topologies with targets encompassing sensitive OAR require sufficient number of beams of 15 or more. For the subgroup of topologies where beam incidences are possible which cover the whole PTV without direct OAR irradiation, the quality dependence on the number of beams is much less pronounced above 9 beams. However, these special non-coplanar beam directions have to be found. On the basis of this work the non-coplanar IMRT techniques can be chosen for further clinical planning studies.

**Electronic supplementary material:**

The online version of this article (doi:10.1186/s13014-015-0494-5) contains supplementary material, which is available to authorized users.

## Background

The aim of this work is to analyze characteristics of non-coplanar IMRT for planning target volumes (PTVs) encompassing an organ at risk (OAR).

### Types of PTV-OAR topologies

**Fig. 1:**
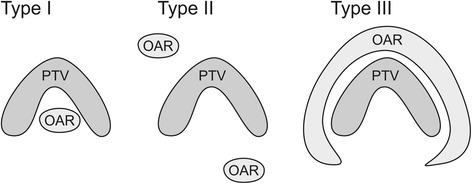
Types of PTV-OAR topologies. Three types of PTV-OAR topologies: type I: PTV (partially) enclosing an OAR; type II OARs loosely distributed around a PTV; type III: one OAR or several OARs encompassing a PTV; contrary to type I, for type II and III the PTV shape needs not to be concave

For intensity modulated radiotherapy, OARs surrounding a convex PTV lead to significantly different solutions than the reverse situation of a PTV encompassing an OAR: Only in the latter case can the OAR be completely spared, if the scatter is neglected (type I geometry, see Fig. 1). An ideal OAR sparing is achieved by the highly inhomogeneous fluence distribution peaking near the beam eye view projection of the OAR [1, 2]. The necessary fluence distribution can be numerically found for arbitrarily complicated PTV-OAR geometries by the inverse planning procedures controlled by the set of dose-volume objectives [3]. For computed tomography (CT) image reconstruction, an incomplete scanning of the Radon space is known to produce aliasing artifacts in image reconstruction. To identify small structures, fine sampling and high resolution is necessary (see i.e. Kak p. 178 [4]). Similar mathematical formalism is also applicable to IMRT-based dose shaping. The generation of deliberately fine textured dose distributions will therefore follow the same rules: Similar to computed tomography, the minor distorted solutions need a sufficient number of projections (beam directions), best in an equidistant manner.

Note that one OAR or several OARs in a dense sequence distributed around a convex PTV cannot in principle be perfectly spared in the coplanar beam arrangement: there are always beam orientations critical for the dose saturation in the PTV, which irradiate through the OARs (type III geometry, Fig. 1). Exact solutions for the inverse planning problem do not exist for the positive definite solution space: negative fluences would be needed. The desired balance between the PTV coverage and the OAR sparing is reflected in the weights of dose-volume objectives used for the inverse planning and has to be controlled by the clinician. The optimal number of beams, their directions and fluence distributions depends on the properties of the OARs. A sparse distribution of OARs around PTV, which allows beam directions which perfectly spare the OAR (type II geometry, Fig. 1) leads to a problem of beam angle optimization (BAO). Often only few beams are needed for high quality plans, if appropriate directions are chosen. In real life, type I, II and III situations are mostly mixed. It should be borne in mind that if non-coplanar directions are allowed, a type I or type III problem can transform to type II. This work is mainly concentrated on type I and type II problems, a PTV encompassing an OAR.

### Non-coplanar techniques vs. coplanar techniques

For type I topologies (PTV encompassing an OAR), it is not yet obvious whether the non-coplanar beam arrangement can improve OAR sparing, as the OAR can be perfectly spared also in the coplanar beam arrangements [1]. An increased sparing of healthy tissue is reported in the IMRT planning study for the simple spherical phantom and a non-coplanar beam arrangement [5]; an increase of the therapeutic width due to improved OAR sparing for the non-coplanar IMRT and a number of clinical cases is reported in [6]. Many compact (eyeballs, lenses, parotid glands, pituitary gland, etc.) or elongated (rectum, urethra, spinal cord, optic nerves) OARs exist that should benefit from non-coplanar IMRT. In the present work, the authors expand the scope of [5] and present the comparison of solutions for more complex phantom topologies and beam constellations. The purpose is to analyze the characteristics of non-coplanar beam incidence versus conventional coplanar IMRT for the type I geometry of PTV encompassing an OAR and to provide simple planning rules for IMRT/intensity modulated arc therapy (IMAT)/volumetric arc therapy (VMAT) planning studies with real patients that will follow. The clinical significance of the observed effects can be evaluated using appropriate biological models for various clinical situations and is beyond the scope of this work.

For type III topologies, the stereotactic treatments using non-coplanar beam arrangements are mostly used. Contrary to a coplanar beam arrangement, additional degrees of freedom of the non-coplanar beam arrangement would in most cases allow better OAR sparing without compromising PTV coverage. At the distances larger than a target diameter away from the target, the dose gradient approaches 1/r^2^ instead of 1/r -behavior for non-coplanar vs. coplanar techniques, respectively. The price for the improved OAR sparing could be longer pathways in the body with additional dose load to the healthy tissue which potentially limits the use of non-coplanar techniques. This aspect of the problem cannot be discussed here, as the solution depends strongly on the actual biological and geometrical parameters.

### Number of beams

The question of the number of beams has to be addressed early: what is the appropriate range of beam numbers to investigate? It is important to note that type I, type II, and type III geometries probably require different number of beams. E.g. for type III geometry, the dose load to surrounding organs can be spread over wider areas and “thinned out”. More available (non-coplanar) beam angles potentially allow better organ sparing, especially of serial organs. For type II geometry, an advantageous beam angle optimization (BAO) can find favorable beam arrangement: even five selected non-coplanar beams achieve the quality of nine equidistant coplanar beams; see e.g. Bangert et al. [7] who investigated type I-like head and neck cases and observed the beam number dependencies that level off between 9 and 11 beams. A good example for a different behavior can be found in Rossi et al. [8]. The authors compare coplanar and non-coplanar techniques for stereotactic body radiotherapy (SBRT) prostate irradiation and observe clear beam number dependences above ten beams. This discrepancy can be understood as follows: Rossi’s prostate cases require clearly concave isodose lines towards rectum and embedded urethra, which we classify as type I geometry. Type I geometry is supposed to require more beams than type II or type III. The larger the OAR radius and smaller the PTV-OAR distance (or the steeper the required dose gradient to the OAR), the more equidistant beams are required for successful dose painting, in analogy to inverse reconstruction methods in computer tomography. Equidistance was proposed by Schreibmann et al. as a class solution for the therapy of prostate carcinoma [9]. As non-coplanar beams are distributed over 2π solid angle (4π if beam entrance and exit are considered), the angular distance between the beams is inherently larger and the advantages of non-coplanar arrangements should become noticeable at least for the number of beams larger then 9–10. Therefore for this study we evaluated plans with 9–16 beams, 16 beams were chosen as an appropriate practical limit, see Results section.

### Number of segments

The question of a sufficient number of segments is restricted to step and shoot IMRT. The number of necessary segments can be estimated based on the pioneering work on inverse planning by Brahme et al. [1]. They discovered the need of a substantial fluence increase near the BEV projection of the OAR to compensate the underdosage in the PTV without simultaneously to compromise the OAR sparing. For an important OAR surrounded by a PTV, the fluence profile featuring such “Brahme-peak” can be realized in practice by two left-sided, two right-sided, and a base segment (S0) [2]. The base segment was termed segment of the order 0, S0, segments which blend the OAR out were called segments of order 1, S1, narrow segments adjacent to the OAR were called segments of order 2, S2. According to this rough guess, up to five segments per beam are recommended.

## Materials and methods

### Materials

A virtual linac Elekta Synergy S with BeamModulator™ multi-leaf-collimator (MLC; “S”), leaf width 4 mm, was commissioned in the therapy planning system (TPS) Pinnacle3™. The optimization engine was “Intensity Modulation™” module available in Pinnacle3™ with 80 steps for pure fluence distribution, and Step and Shoot Direct Machine Parameter Optimization (DMPO™) with two runs 40 steps each for DMPO, thereof ten steps for fluence optimization before sequencing. For DMPO, the total number of segments is given and it is up to a sequencer to distribute the segments among the beams. The resulting fluence distributions were investigated qualitatively.

The gradient based optimization algorithm is based on the composite objective value (COV) as objective function. It sums weighted relative quadratic deviations from the clinical dose-volume requirements. After the optimization procedure, the dose is normalized to the mean dose in the PTV (resulting in monitor unit (MU) corrections of less than 0.5 %).

### Research topics

**Fig. 2:**
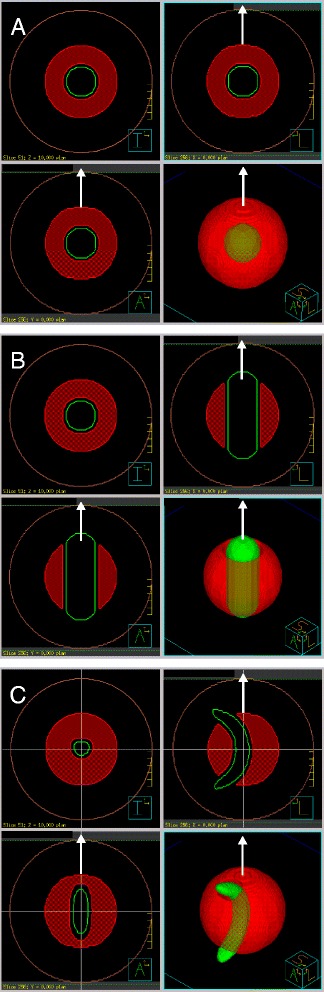
PTV-OAR topologies used in the study. Shown are for each OAR shape ball (A), cylinder with rounded ends (B), banana (C): three orthogonal planes through the phantom center and a perspective view of the green OAR embedded in the transparent red PTV. The OAR in B, C has inferior (I) - superior (S) orientation, with its longer dimension parallel to the table (white arrow). Brown line shows the phantom surface

The purpose of this work was to investigate capabilities of IMRT with non-coplanar beams. Following parameters were varied for systematic studies:

- OAR shape (Fig. 2):

A) spherically symmetric (spherical),
B) axially symmetric (cylindrical),
C) mirror symmetric (banana-shaped)

**Fig. 3:**
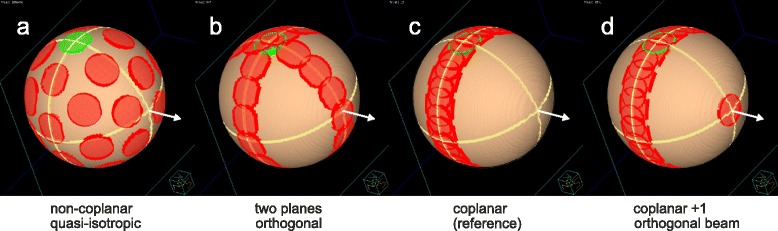
Beam port constellation. Shown are the beam entrance ports (smaller circles) and exit ports (larger circles) on a spherical phantom for a ball shaped target (for simplicity). Beam arrangements for the techniques (**a**) Q4π, non-coplanar, quasi-isotropic; (**b**) 2P, split into two planes; (**c**) Co coplanar; (**d**) Co + 1 coplanar plus additional orthogonal beam. Always the cases with highest beam numbers are depicted (**b**, **c**: 15; **a**, **d**: 16); green: entrance port of the [gantry 0°; table 0°] beam. White arrow: table axis for standard orientation of the beam set

- irradiation technique (Fig. 3):

- non-coplanar quasi-isotropic,
- coplanar split in two orthogonal planes,
- coplanar equidistant,
- coplanar equidistant plus one additional orthogonal beam.

For fluence optimization, the relative orientation of the beams was maintained, and the orientation of the whole set of beams with respect to the PTV-OAR arrangement was varied for the OAR shapes B and C to study the effect of the particular beam set orientation on the plan quality. Taking symmetries into account, up to 58 orientations were systematically investigated to allow an estimation of lowest and highest COV for each technique in any orientation. Dense beam axis positioning with small angles (3° or 5° for high and low number of beams, respectively) with respect to the OAR axis was especially examined to find the overall minimal COV.

OARs, PTVs, and irradiation techniques are described below in more detail. Plan quality is conjectured to depend strongly on:

- number of beams, therefore for each plan 2–3 practical number of beams, between 9 and 16, was chosen: **a**: 10, 16; **b**, **c**: 9, 11, 15; **d**: 10, 12, 16; (always highly symmetric configurations)
- number of segments, therefore for each beam arrangement optimal fluence distribution (no sequencing), “large” (120) and “small” (64) number of segments were considered.

Gantry and table angles for the 15 and 16 beam technique variants are presented in Table 1.

**Table 1:** Beam arrangements

a		b		c		d	
Table angle	Gantry angle	Table angle	Gantry angle	Table angle	Gantry angle	Table angle	Gantry angle
0°	0°	0°	0°	0°	0°	0°	0°
0°	37°	0°	24°	0°	24°	0°	24°
0°	79°	0°	48°	0°	48°	0°	48°
0°	117°	0°	72°	0°	72°	0°	72°
36°	243°	0°	96°	0°	96°	0°	96°
36°	281°	0°	120°	0°	120°	0°	120°
36°	323°	0°	144°	0°	144°	0°	144°
72°	217°	0°	168°	0°	168°	0°	168°
72°	259°	90°	192°	0°	192°	0°	192°
72°	297°	90°	216°	0°	216°	0°	216°
324°	63°	90°	240°	0°	240°	0°	240°
324°	101°	90°	264°	0°	264°	0°	264°
324°	143°	90°	288°	0°	288°	0°	288°
288°	37°	90°	312°	0°	312°	0°	312°
288°	79°	90°	336°	0°	336°	0°	336°
288°	117°					270°	90°

The following parameters were - not independently - varied for completeness (only for sequenced techniques):

- set of objectives which tend to achieve more PTV coverage or more OAR sparing: PTV-conformal (15 objectives) or OAR-sparing (22 objectives), respectively,
- dose grid 2 mm / 4 mm,
- collimator angle 0°/20° (K00, K20, planned with Pinnacle3™ V 9.0 and 9.2 respectively).

The following combinations were chosen: K20_2mm_PTV-conformal, K00_4mm_PTV-conformal, K00_4mm_OAR-sparing.

For more extensive exploration of different orientations of the beam sets, only K00_4mm_PTV-conformal was used.

### PTV-OAR topologies

The OAR shapes are motivated by the clinical cases mentioned in the introduction. Three types of OAR shapes shortly described above were embedded in a spherical PTV, 10 cm in diameter, centered in a ball shaped phantom, 20 cm in diameter. The outer shape is chosen to be as regular as possible, not to mask the effect of embedded organs. The OARs were inflated by 5 mm in all directions to define the cut-out of the PTV. The following OAR shapes were considered (Fig. 2):

A) ball of diameter 3 cm;
B) cylinder of diameter 3 cm oriented parallel to the table axis with rounded ends, penetrating the PTV;
C) banana-shaped OAR of diameter 2 cm and bending radius 5 cm, penetrating the PTV.

In contrast to the cylindrical geometry of case B, for the cases A and C there is no gantry angle that would allow complete irradiation of the PTV combined with complete blocking of the OAR. It should be mentioned that A and C always lead to type I situations, also when switching from coplanar to non-coplanar techniques. Case B, however, becomes type II-like for a non-coplanar technique: it is obvious to direct one of the beams along the cylinder axis for complete OAR blending. All elongated OARs were parallel to the table axis, similar to typical clinical objects (rectum, spinal cord).

For each PTV-OAR topology from Fig. 2 several plans with 2–3 practical numbers of beams were created in each of the following categories (see Fig. 3 and Table 1):

- Non-coplanar quasi-isotropic (Non-Co): A quasi-isotropic arrangement of 16 beams of triakis icosahedral symmetry (a combination of icosahedral and dodecahedral solids) [5] resulting in angular distances between the beams of 63°, 42° and 37°. It was compared to a dodecahedral combination of 10 fields alone. Both highly symmetric beam arrangements can be applied using only five different table angles (which is advantageous, since table rotations are time-consuming).
- Field axes in two orthogonal planes each (2P): 9, 11, and 15 beams arranged in two equidistant coplanar sets, with half of beams applied perpendicular to the principal plane via table rotation to 90°.
- Coplanar equidistant (Co): classical coplanar technique with equidistant 9, 11 and 15 beams (reference).
- Coplanar equidistant supplemented by a single orthogonal beam along the table axis (Co + 1).

The number of beams was chosen to allow a high degree of symmetry. An entrance port of a beam never coincided with an exit port of another beam. All techniques cover maximally one hemisphere; the substitution of a beam by an opposed beam in all examined samples did not essentially influence the results. Except technique **a**, all others are suitable for irradiation of the trunk. For each combination of PTV-OAR topology and beam arrangement, the dependence of the plan quality indices on the number of beams was investigated for pure fluence distribution, “large”, and “small” number of segments. Technique **a** (Non-Co) was motivated by the question, whether many non-coplanar beam directions routinely used for standard stereotactic radiotherapy of type II and type III geometries are also useful in the reverse situation of the OAR surrounded by the target. The motivation behind the orthogonal beam sets of the technique **b** (2P) was to avoid the shadow zone of the cone beam geometries in the Radon space and to satisfy the Tuy-Smith condition (see e.g. Buzug [10] pp. 368–378 or [11]). The technique **c** (Co) was chosen as a reference. Technique **d** (Co + 1) can be regarded as an intermediate technique between **b** and **c**.

### Quality indicators

We evaluated plan quality of optimized plans in different ways. First, overall plan quality can be characterized by the composite objective value (COV) at the end of optimization. COV is the sum of all weighted objective values as described above. Objective values are defined as volume-normalized quadratic penalties determined for points in the dose volume histograms (DVHs). The lower the COV for a certain set of objectives, the better the beam/segment arrangement fulfills the dose requirements expressed through this set of objectives. Due to the high number of objectives (15 and 22, respectively), the deviation from the desired DVH shape is adequately represented by the residual COV at the end of optimization. COV is thus a plan quality indicator as long as all relevant DVH parameters are reflected in the objectives [12]. For the purpose of this study we used COV normalized to the best (lowest) COV among all techniques for a certain topology, *nCOV*.

Besides *nCOV* two indices were used to measure the overall plan quality in PTV and OAR:

- quality index *S*_*D*_, sum of violations of dose requirements for PTV and OAR [13]. For the purpose of this study *S*_*D*_ index considered violations of the following requirements: PTV V_95%_ ≥ 99 %; PTV V_105%_ ≤ 5 %; OAR V_70%_ ≤ 1 %; healthy tissue (whole phantom, PTV excluded) HT V_80%_ ≤ 15 %; HT V_100%_ ≤ 2 %; HT D_max_ ≤ 105 % . *S*_*D*_ = 0 if all above conditions are fulfilled.
- Conformity number *CN* [14] for reference isodose *D*_*RI*_ = 95 %; defined as *CN := (TV*_*RI*_ *)* ^*2*^ */(V*_*RI*_ *TV)*

for target volume *TV*, part of target volume surrounded by the reference isodoses *RI*, *TV*_*RI*_, and total volume *V*_*RI*_ surrounded by the reference isodose.

Additionally, the necessary monitor units *MU* and the mean dose to the healthy tissue, *HT D*_*mean*_, were evaluated.

## Results

### Qualitative analysis of beam fluence

**Fig. 4:**
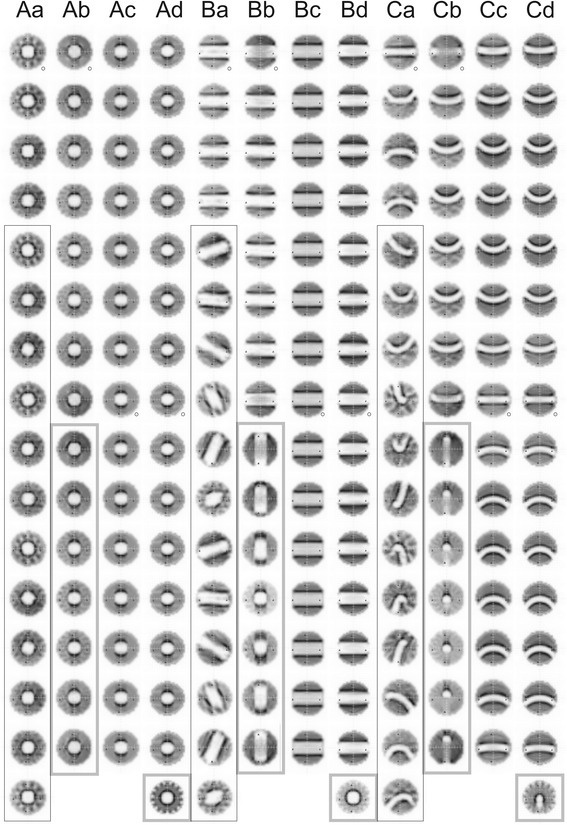
Typical fluences for non-coplanar and coplanar techniques. (A) spherical OAR; (B) cylindrical OAR; (C) banana-shaped OAR; (**a**) Quasi-isotropic technique Q4π (16 beams), (**b**) beams arranged in two orthogonal planes 2P (15 beams); (**c**) coplanar technique Co (15 beams); (**d**) coplanar + 1 non-coplanar beam Co + 1 (16 beams); o: table angle = 0° ^ gantry angle = 0°; grey thick bordered box: table angle = 90°; black thin bordered box: table angle ≠ 90° ^ ≠ 0°

Figure 4 shows optimized fluence distributions for different combinations of PTV-OAR topology and irradiation technique in standard orientation (see Fig. 3). Substructures of Fig. 4 can be viewed in more detail in the Additional file 1. The fluence Brahme-peak [1, 2, 5] adjacent to the OAR projection could clearly be seen as a dark shaded area parallel to the rotation axes of the gantry, see e.g. Fig. 4Ba. Technique **b** uses two orthogonal rotation axes, so for OAR B (see Fig. 4Bb), the fluence at table angle 90° included Brahme peaks also above and below the cylinder. For more isotropic irradiation (technique **a**) (Fig. 4Aa), Brahme peak surrounded the OAR [5]. For non-coplanar techniques **a**, **b** and **d** substructures of the fluence distribution reflected the symmetry of the beam arrangement. E.g. for Aa five table angles resulted in a C_10_-symmetry-like fluence structure. Co + 1 technique **d** (Fig. 4Ad) generated substructures in the coplanar fields: parallel stripes of reduced fluence tangential to the OAR along the direction of the non-coplanar beam. The substructures in the fluence of the non-coplanar field itself (Fig. 4Ad, lowest subfigure) reflected 15 equidistant coplanar fields.

We do not show fluences for different numbers of beams, in conclusion, more beams led to finer and less intense modulations; the maximal number of beams, 16, was chosen as a practical compromise. The non-coplanar technique **a** in the limiting case of infinitely many beams could be considered as a superposition of infinite number of coplanar irradiations around isotropical distributed rotation axes. For this case, all substructures except the Brahme peak wiped out. For a non-coplanar technique with limited number of beams substructures in the fluence distribution could not be avoided. Note, that Brahme peak required for non-coplanar IMRT is narrower than that for coplanar IMRT, as was observed in [5].

### Plan quality for different beam sets and beam set orientations

**Fig. 5:**
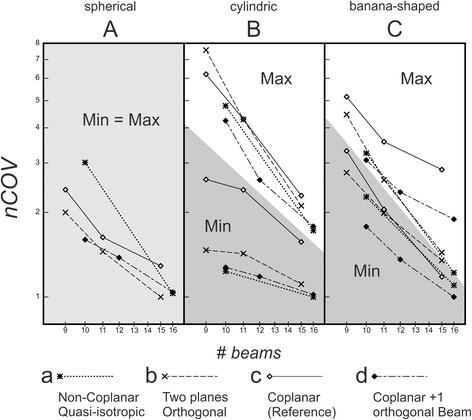
Range of nCOV for varying beam set orientation. Topology (B, C): The maximum (Max: white area) and minimum (Min: dark grey area) normalized composite objective value *nCOV* for varying beam set orientations vs. number of beams (*# beams*) in double logarithmic scale. (A) due to spherical symmetry all beam set orientations are equivalent (light grey). The plans (techniques **a, b, c, d**) were fluence optimized. The lines are for eye guiding only

For each irradiation technique **a, b, c, d**, the minimum (Min) and the maximum (Max) *nCOV* value were determined for all possible beam set orientations (Fig. 5).

### Characteristics of the different topologies

For spherical symmetric geometry A, Min and Max were identical: there is no preferred beam set orientation for any irradiation technique. For cylindrical geometry B, Min and Max differed the most among all non-coplanar techniques. In addition, whereas for all geometries an obvious beam number dependence of Min was observed, Min for cylindrically symmetric geometry B depended only weakly on the number of beams for all non-coplanar techniques. The minimum of *nCOV* for geometry B always occurred when one of the beams was precisely incident along the cylinder axis. This is a pure type II situation: such a beam spares the OAR completely without compromising PTV coverage, the increased number of beams does not improve plan quality much. For the “bent” banana-shaped geometry C the preferred beam arrangement corresponding to Min is less obvious, the number of beams plays more role: the dependence of Min on the number of beams is more pronounced.

### Best choice of technique

**Fig. 6:**
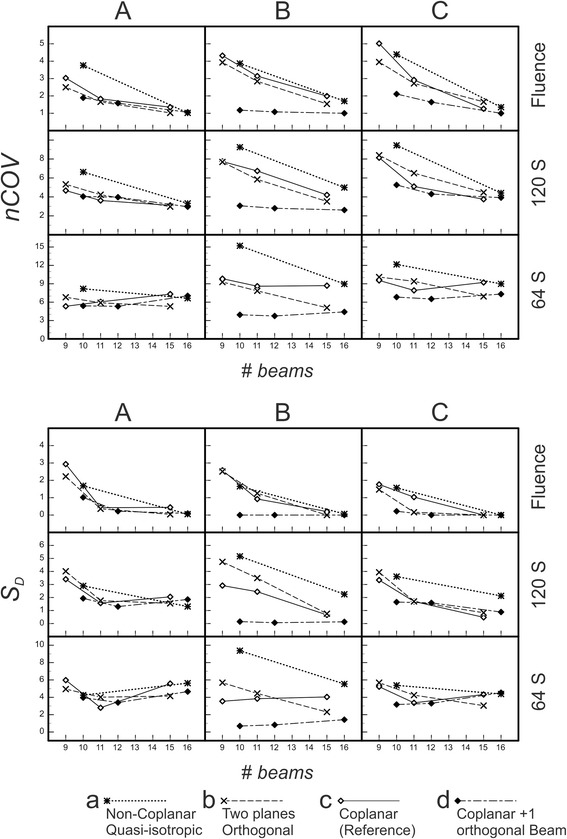
*nCOV* and *S*_*D*_. The normalized composite objective value *nCOV* (*top*) and the quality index *S*_*D*_ (*bottom*) for topologies A, B, C vs. number of beams (*# beams*). (A) spherical OAR; (B) cylindrical OAR; (C) banana-shaped OAR. The plans were optimized using pure fluence optimization (Fluence) and DMPO for maximum 120 segments (120 S) and 64 segments (64 S), respectively. Compared are the results from four techniques: (**a**) quasi-isotropic non-coplanar (Q4π), (**b**) beams arranged in two orthogonal planes (2P), (**c**) coplanar (Co; reference), (**d**) coplanar + 1 orthogonal beam (Co + 1)

For non-type II topologies the number of beams was more important than the choice of technique. Techniques **a** and **d** both provide good results. The quasi-isotropic technique **a** was less sensitive with respect to the orientation of the beam set; however, this technique was very sensitive to changes of the beam number and demands 16 beams (or more). The Co + 1 technique **d** would be the most relevant technique, if the number of beams is restricted to about ten, see Figs. 5 and 6. Also, the 2P-technique **b** or Co + 1 **d** always increased the plan quality with respect to the coplanar situation at a comparable number of beams.

### Plan quality for different numbers of segments

To obtain linac deliverable plans, the ideal fluences were sequenced into segments which were then run through DMPO algorithm. For each technique only one beam set orientation was chosen for sequencing.

As the quality of fluence-optimized plans were largely independent from the combination of the collimator angle and the set of dose objectives (K20_PTV-conformal, K00_PTV-conformal, K00_OAR-sparing), only the mean values over three constellations are presented. The dependences of quality indices *nCOV* and *S*_*D*_ on number of beams for chosen number of segments are shown in Fig. 6; they show the same trends. The *CN* index is not depicted, as its dependence was similar to that of *nCOV* and *S*_*D*_, but less pronounced. For all techniques with 120 segments the observable trends were qualitatively the same as for fluence based plans: both *COV* and *S*_*D*_ indices decreased for increasing number of beams; the plan quality improved. The quality improvement was most pronounced for the quasi-isotropic non-coplanar technique **a**. For the smaller numbers of beams and segments, the quality of the technique decreased. Note, that for 64 segments, a minimum *S*_*D*_ occurred for the number of beams 11–12, i.e. at about five segments per beam as expected (see Background section). For the larger number of beams, i.e. smaller number of allowed segments per beam, the plan quality degraded. The authors suppose a problem with the DMPO sequencer in Pinnacle3™, which does not allow to distribute different segment orders to neighboring beams for decreased number of segments per beam as would be necessary ideally [15]. Such behavior is not expected for optimization algorithms like simulated annealing [16].

### Monitor units and healthy tissue

**Fig. 7:**
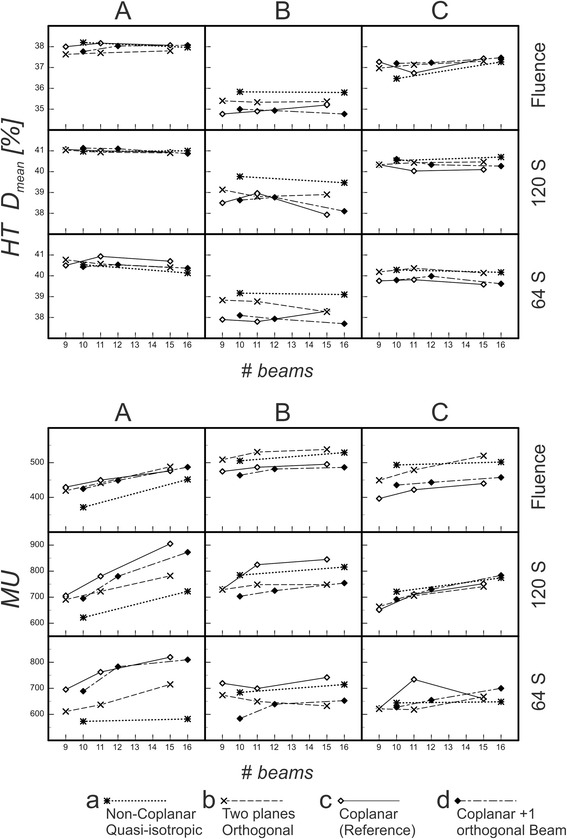
*HT D*_*mean*_ and *MU.* The mean dose to the healthy tissue *HT D*_*mean*_ (top) and the necessary monitor units *MU* (bottom) for topologies A, B, C vs. number of beams (*# beams*). (A) spherical OAR; (B) cylindrical OAR; (C) banana-shaped OAR. The plans were optimized using pure fluence optimization (Fluence) and DMPO for maximum 120 segments (120 S) and 64 segments (64 S), respectively. Compared are the results from four techniques: (**a**) quasi-isotropic non-coplanar (Q4π), (**b**) beams arranged in two orthogonal planes (2P), (**c**) coplanar (Co; reference), (**d**) coplanar + 1 orthogonal beam (Co + 1)

We observed an increasing MU number for larger number of beams (Fig. 7b): always for 120 segments (typically around 3 % more MU for each additional beam), mostly for 64 segments. Plans using fewer segments needed less MU. However, the mean dose to the healthy tissue did not increase correspondingly (Fig. 7a). Smaller segment areas compensated the increased number of monitor units. *HT D*_*mean*_ was almost independent from the beam arrangement for type I topologies A and C.

## Discussion

The important observation is a clear dependence of the plan quality on the number of beams even above 9 to 11 beams, which is in agreement with results of other studies [8] for type I topologies like A and C. The effect of beam number is even more important than the choice of technique. Better dose painting results for the larger number of radiotherapy beams can be understood as an inverse analogy to the CT technique, where small angular steps between image acquisitions ensure small “reconstruction errors” (aliasing artifacts) [3, 17]. It remains to be investigated whether this effect is recognizable for the dense series of gantry angles of VMAT - provided the used optimization algorithm supports these potential abilities of VMAT. The fluence-optimized two-plane technique **b** was always better than the beam-number-equivalent coplanar technique **c**. Whether this behavior can be traced back to a better coverage of the Radon space [10] could be tested using larger and smaller targets and OARs, leading to larger or smaller aperture angles of the beams and - corresponding - less or more coverage.

However, the situation in case B is quite different. Here the target and the OAR do not overlap in the eye view of the beam parallel to the common axis of OAR and PTV. So, PTV can be covered from this direction without OAR sparing to be compromised. This is a typical type II situation, where a good choice of a few beam directions can be more effective than an increased number of beams, and no additional beam can add serious benefits. Thus, in Fig. 6b, the quality indices *nCOV* and *S*_*D*_ are nearly independent from the number of beams in the studied range for “well chosen” beam arrangement **d**. If such a “preferred” beam direction is not obvious, a beam angle optimization may be necessary.

The three types of PTV-OAR topology of Fig. 1 help to systematize the choice of technique for daily work. This choice follows from the presented planning study and is in good agreement with previous studies.

In particular, Popple et al. [18] consider convex breast and mediastinal tumors which according to our classification fall into type II. The authors report that no more then 6–7 beams (11 beams for the most complicated head case) are needed for adequate PTV coverage/OAR sparing. Wang et al. [19] compare 5 beam BAO against 9 beam coplanar IMRT for two groups of paranasal sinus carcinoma cases, with one group characterized by the clear separation of PTV from OAR, typical for type II topology. This group profited most from 5 beam BAO, while the other group showed better results from conventional coplanar 9 beam IMRT treatment. Bangert et al. [7] report on a similar group of tumor localizations; a distinction like that of Wang is not made, however, the quality of their 11 coplanar beam plans is similar to that of optimized non-coplanar five-beam technique. Note, that these results are in good agreement with our finding that a non-coplanar technique can be “caught up” by a conventional coplanar technique with large enough number of beams, if enough segments are allowed, see Fig. 6B. For the type III topology, Dong et al. [20] report optimal number of beams between 10 and 22 for an optimized quasi-isotropic non-coplanar beam arrangement similar to our Q4π technique **a**. The high beam number probably reflects the need of steep dose gradients towards the surrounding tissue. However, type III was not the main focus of this work.

Perhaps due to principal optimization problems in BAO [21] and corresponding combinatorial problems of the concurring beam angle selection (BAS) method [7], only a limited number of sources is available. Mostly only low non-coplanar beam numbers were used (e.g. [22, 23]), below the range of our study.

For type I topology, a PTV partially enclosing an OAR, a sufficient number of beam directions (15 or more) is more important than the choice of technique. However, if such “good” OAR-sparing beam direction could be found, BAO becomes more important: for OAR structures that can be spared using selected beam directions (type II topology), a beam incidence along that direction is necessary; the total number of beams can then be reduced without considerable quality loss. If no “good” direction can be found and no BAO is available, for OAR-sensitive irradiations in the head the quasi-isotropic technique with 16 beams in five table orientations is preferable; for Pinnacle3™ DMPO a sufficient number of segments has to be allowed for sequencing.

A technique with only one beam or half of beams applied perpendicular to the principal plane provides stable results of high quality for practical number of beams (9–16) and segments (under 120) potentially even in the case of trunk applications. The Co + 1 technique is recommended especially for Pinnacle DMPO if few segments are allowed. Note that only one additional table rotation is required for both techniques, and that the techniques can be partially applied as IMAT/VMAT. As more gantry angles improve target coverage and OAR sparing, a combination of non-coplanar technique with IMAT/VMAT seems promising.

## Conclusions

The purpose of the present work is to study potentials of non-coplanar IMRT technique using schematic phantom cases which represent generalized OAR – PTV geometries. The work provides the basis for further planning studies with real clinical cases. The results, however, should not be applied to highly individual clinical cases without consideration.

In conclusion, if the extensive BAO is not available or meaningful, three types of PTV-OAR topology introduced in the present study provide a good indicator for the choice between coplanar and non-coplanar beam arrangement and the choice of particular non-coplanar technique. The relative comparison of considered non-coplanar techniques to the reference coplanar technique is shown in Table 2. A good result (+ or ++) in a “Min” column means: If the best orientation of the beam set can be evaluated (using BAO), this technique is to be preferred with respect to the coplanar technique. A good result (+ or ++) in a “Max” column means: the most unfavourable result for the considered technique was better than the most unfavourable result for the coplanar reference technique; it could be preferable, if no BAO is available.

**Table 2:** Comparison of non-coplanar techniques to the reference coplanar technique

		Type I							Type II	
		Fluence (Fig. 5)			120 segments (Fig. 6a)		64 segments (Fig. 6a)		Fluence (Fig. 5)	
		A	C		A	C	A	C	B	
			Min	Max					Min	Max
9-10 beams	a	−	+	++	− −	− −	− −	− −	++	+
	b	+	+	+	−	−	−	Ο	+(+)	−
	c	Ο	Ο	Ο	Ο	Ο	Ο	Ο	Ο	Ο
	d	++	++	++	Ο	++	Ο	++	++	++
15-16 beams	a	+	+	++	Ο	Ο	Ο	Ο	++	++
	b	++	−	+(+)	Ο	Ο	+	+	+(+)	+
	c	Ο	Ο	Ο	Ο	Ο	Ο	Ο	Ο	Ο
	d	+	++	+	Ο	Ο	+	+	++	++

Recommendations for the choice of technique, following Figs. 5 and 6, for types of PTV-OAR-topologies (Fig. 1)

o equivalent, + better, ++ best choice, etc

Techniques/beam sets:

a) quasi-isotropic non-coplanar (Q4π)

b) beams arranged in two orthogonal planes (2P)

c) coplanar (Co; reference)

d) coplanar + 1 orthogonal beam (Co + 1)

“Min” refers to best and “Max” to the most unfavourable orientation of a given beam set (a, b, c, or d), respectively

(++ in the “Max” column means: the worst result for the considered technique was much better than the most unfavourable result for the reference technique)

For pure type I problems (like A and C) in the head, ideally use a non-coplanar quasi-isotropic technique **a** (Q4π) with 15 or more beams and many segments (100+) or sliding window technique. If less beams or less segments are allowed, it seems more practical to use **d** (Co + 1), the coplanar technique with additional orthogonal beam. For less segments also technique **b** (2P) with coplanar beams split regularly into two planes is an option. Techniques similar to **b** and **d** could also be applied in the trunk.

For type II topologies, also quasi-isotropic non-coplanar technique **a** is recommended for the head, especially if no BAO can be performed or preferred beam directions are not obvious. For limited BAO computational time it could serve as starting constellation. A good choice is technique **d**, coplanar with one or several nearly orthogonal beams along potentially “good” axes which allow PTV coverage without OAR sparing to be compromised. The number of coplanar beams can then be reduced without appreciable quality loss. This technique can also be applied for trunk irradiations.

Type III topologies were not the topic of this work; however, for OARs encompassing a PTV a technique with minimum overlap between the beams should be preferred, to decrease maximum dose to the OAR, albeit at the expense of healthy tissue. This is again the case for the quasi-isotropic non-coplanar technique **a**, which can also provide a good starting point for BAO (which is needed for local beam angle optimization [23]).

The future planning studies will concentrate on real clinical cases. The PTV-OAR topologies will be grouped by their type (I, or type II, or III), the practical strategy for daily non-coplanar planning will be further developed, and the class solutions for standard clinical cases will be created. Planning studies are under way to test the presented recommendations.

## Additional file

Additional file 1:
**Typical fluences for non-coplanar and coplanar techniques.** A: spherical OAR; B: cylindrical OAR; C: banana-shaped OAR; **a**: Quasi-isotropic technique Q4π (16 beams), **b**: beams arranged in two orthogonal planes 2P (15 beams); **c**: coplanar technique Co (15 beams); **d**: coplanar + 1 non-coplanar beam Co + 1 (16 beams); o: table angle = 0°^ gantry angle = 0°; grey thick bordered box: table angle = 90°; black thin bordered box: table angle ≠ 90° ^ ≠ 0°. (PNG 10614 kb)

## Abbreviations

2P: Two orthogonal planes (planning technique)
# beams: Number of beams
BAO: Beam angle optimization
BAS: Beam angle selection
CN: Conformity number
Co: Coplanar equidistant (planning technique)
Co + 1: “Co” supplemented by a single orthogonal beam (planning technique)
COV: Composite objective value
DMPO: Direct machine parameter optimization
DVH: Dose volume histograms
IMAT: Intensity modulated arc therapy
IMRT: Intensity modulated radiation therapy
MLC: Multi-leaf-collimator
MU: Monitor units
nCOV: Normalized composite objective value
OAR: Organ at risk
PTV: Planning target volume
Q4π: Non-coplanar quasi-isotropic (planning technique)
SBRT: Stereotactic body radiotherapy
S_D_: Quality index
TPS: Therapy planning system
TV: Target volume
TV_RI_: Part of target volume surrounded by the reference isodose RI
VMAT: Volumetric arc therapy
V_RI_: Total volume surrounded by the reference isodose RI
