# Supplementary figures and images for: Characteristics of non-coplanar IMRT in the presence of target-embedded organs at risk
Source: Radiat Oncol. 2015 Oct 12;10:207. doi: 10.1186/s13014-015-0494-5 (PMC5480416; doi:10.1186/s13014-015-0494-5)

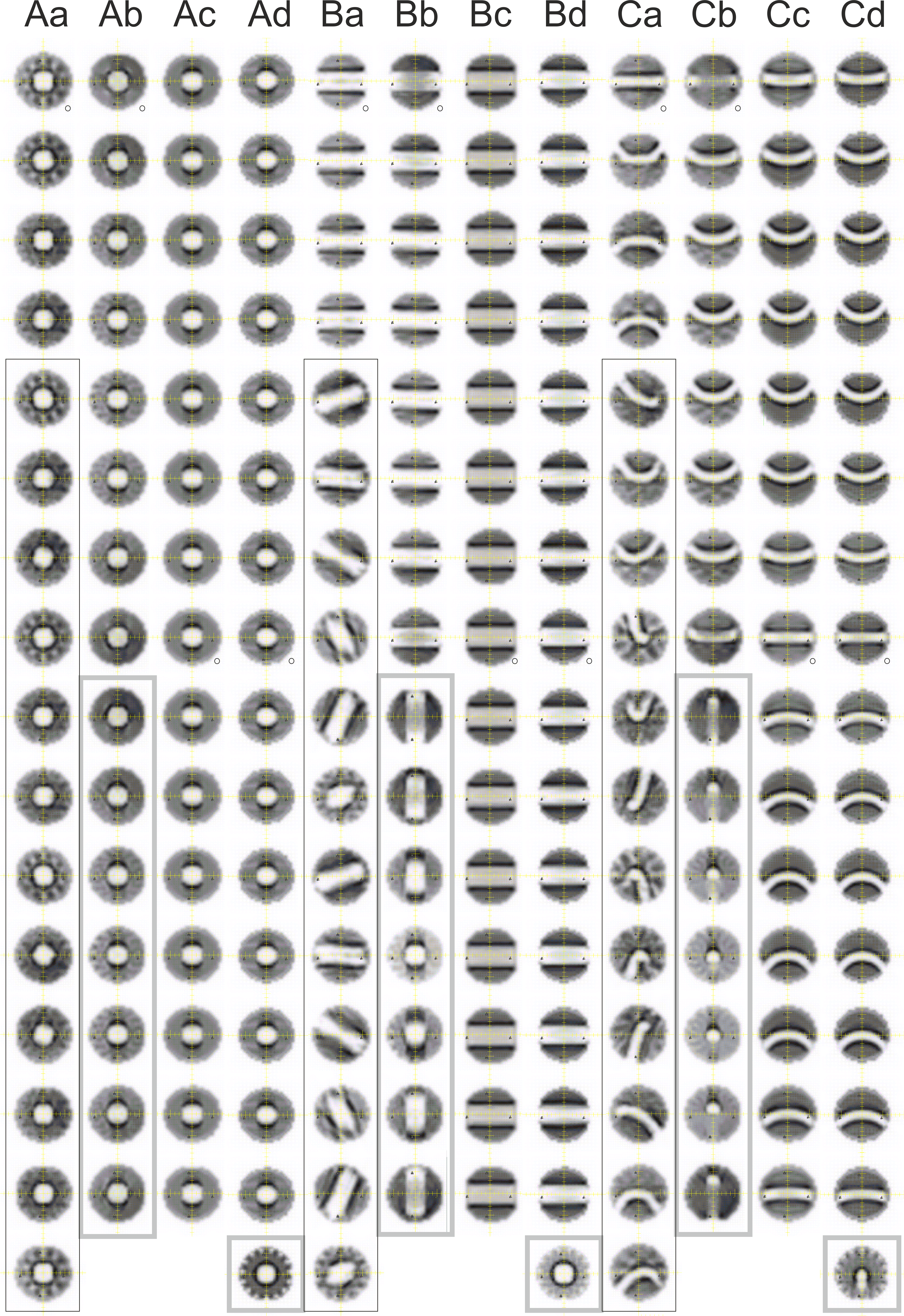

Supplement: Additional file 1: — Typical fluences for non-coplanar and coplanar techniques. A: spherical OAR; B: cylindrical OAR; C: banana-shaped OAR; a: Quasi-isotropic technique Q4π (16 beams), b: beams arranged in two orthogonal planes 2P (15 beams); c: coplanar technique Co (15 beams); d: coplanar + 1 non-coplanar beam Co + 1 (16 beams); o: table angle = 0°^ gantry angle = 0°; grey thick bordered box: table angle = 90°; black thin bordered box: table angle ≠ 90° ^ ≠ 0°. (PNG 10614 kb) [file 13014_2015_494_MOESM1_ESM.png]
